# Boosting Sodium Storage of Fe_1−*x*_S/MoS_2_ Composite via Heterointerface Engineering

**DOI:** 10.1007/s40820-019-0311-z

**Published:** 2019-09-23

**Authors:** Song Chen, Shaozhuan Huang, Junping Hu, Shuang Fan, Yang Shang, Mei Er Pam, Xiaoxia Li, Ye Wang, Tingting Xu, Yumeng Shi, Hui Ying Yang

**Affiliations:** 1 International Collaborative Laboratory of 2D Materials for Optoelectronics Science and Technology of Ministry of Education, College of Optoelectronic Engineering, Shenzhen University, Shenzhen, 518060 People’s Republic of China; 2 Pillar of Engineering Product Development, Singapore University of Technology and Design, 8 Somapah Road, Singapore, 487372 Singapore; 3 Engineering Technology Research Center for 2D Material Information Function Devices and Systems of Guangdong Province, College of Optoelectronic Engineering, Shenzhen University, Shenzhen, 518060 People’s Republic of China; 4 Key Laboratory of Material Physics of Ministry of Education, School of Physics and Engineering, Zhengzhou University, Zhengzhou, 450052 People’s Republic of China

**Keywords:** Heterostructure, Heterointerface, Diffusion barrier, Ion reservoir, Sodium ion battery

## Abstract

Improving the cycling stability of metal sulfide-based anode materials at high rate is of great significance for advanced sodium ion batteries. However, the sluggish reaction kinetics is a big obstacle for the development of high-performance sodium storage electrodes. Herein, we have rationally engineered the heterointerface by designing the Fe_1−*x*_S/MoS_2_ heterostructure with abundant “ion reservoir” to endow the electrode with excellent cycling stability and rate capability, which is proved by a series of in and ex situ electrochemical investigations. Density functional theory calculations further reveal that the heterointerface greatly decreases sodium ion diffusion barrier and facilitates charge-transfer kinetics. Our present findings not only provide a deep analysis on the correlation between the structure and performance, but also draw inspiration for rational heterointerface engineering toward the next-generation high-performance energy storage devices.

**Electronic supplementary material:**

The online version of this article (10.1007/s40820-019-0311-z) contains supplementary material, which is available to authorized users.

## Highlights

- Fe_1−*x*_S/MoS_2_ heterostructure with abundant “ion reservoir” interfaces is designed to reduce sodium ion diffusion barrier and facilitate charge-transfer kinetics, thus endowing the electrode with excellent cycling stability and rate capability.
- The in-depth analysis on the dynamic relationship between heterointerface and sodium storage performance carves a new path for interface engineering toward the next-generation high-performance energy storage devices.

**Electronic supplementary material:**

The online version of this article (10.1007/s40820-019-0311-z) contains supplementary material, which is available to authorized users.

## Graphical abstract

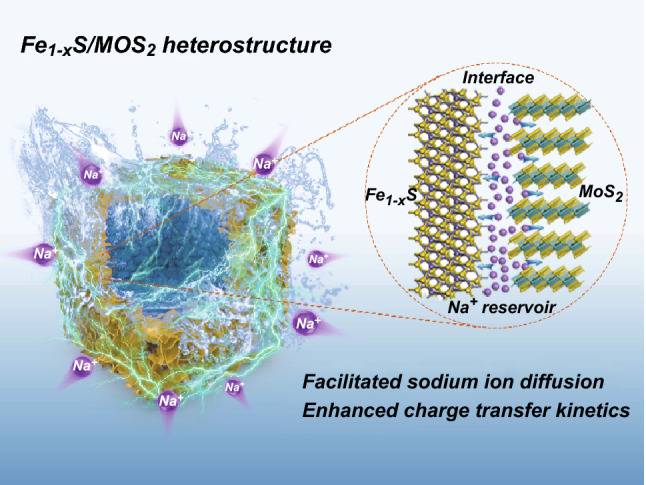

## Introduction

Sodium ion batteries (SIBs) have aroused great interests as a promising substitute to conventional lithium ion batteries (LIBs) owing to the overwhelming superiority in low cost and abundant natural resources [1–4]. However, the sluggish reaction kinetics induced by the larger ionic radius of Na ions deteriorates their electrochemical properties seriously [5, 6]. The key point to achieve high-performance SIBs relies on the rational design of electrode materials with satisfactory Na ions insertion/extraction performance during repeated cycles, especially for the active anode host materials. Among the various anode materials, transition metal sulfides (M_*x*_S_*y*_) have drawn extensive attentions due to their high sodium storage capacity [7–10]. Moreover, the weaker M–S bond than M–O bond is more kinetically favorable for electrochemical conversion reaction, leading to a better redox kinetics and reversibility [11]. As a typical example, iron sulfide (Fe_1−*x*_S) has risen to prominence because of its high theoretical capacity (~ 610 mAh g^−1^), natural abundance and environmental benignity [12, 13]. Nonetheless, there are still obstacles to its commercial applications mainly due to the poor intrinsic conductivity and severe volume changes during the sodiation/desodiation processes, thus resulting in inferior cycling stability and rate capability [14]. Besides Fe_1−*x*_S, 2D layered molybdenum disulfide (MoS_2_) is another promising anode candidate for SIBs due to its analogous structures to graphite [15–17]. In particular, the large interlayer spacing (0.62 nm) and high mechanical strength of MoS_2_ are able to facilitate Na^+^ migration and alleviate structural deformation during the discharge/charge processes [18]. Considering the advantages of these two metal sulfides, a hybrid composite by combining Fe_1−*x*_S with MoS_2_ can be rationally designed and fabricated to expectedly achieve the desirable performance.

Constructing unique heterostructures to create diverse interface effects offers unprecedented opportunities in various fields, such as solar cells, photocatalysis and electrocatalysis [19–27]. The heterointerfaces deriving from coupling nanostructures with different properties can greatly accelerate charge transport and improve reaction kinetics [28]. Nishitani et al. [29] have studied the charge-transfer effects in CdO/SnTe heterointerfaces, which revealed a large fourfold enhancement of electron mobility. Yin et al. [30] have investigated the charge carrier transfer in the bulk heterostructures and indicated the balanced hole/electron mobilities are key factors to attribute high solar cell performance. Hints from the previous reports indicate that rational interface designing could be of great importance for the improvement of conversion reaction kinetics in rechargeable SIBs. Some emerging heterostructures such as Ni_3_S_2_/MoS_2_, Sb_2_S_3_/SnS_2_ and Sb_2_S_3_/MoS_2_ have demonstrated outstanding electrochemical performance, which is difficult to realize in a signal material system [31–36]. However, the detailed synergistic effects and heterointerface properties are rarely revealed.

Herein, we design a Fe_1−*x*_S/MoS_2_ heterostructure with abundant “ion reservoir” to provide fast Na^+^ diffusion channels and interpret specifically the correlation between heterointerface and sodium ion diffusion. The DFT calculations further confirm that the heterointerface significantly reduces the diffusion barrier and facilitates the charge-transfer kinetics, hence enabling excellent cycling stability and rate capability. Our findings not only provide in-depth understanding of the dynamic relationship between heterointerface and electrochemical performance, but also carve a new path for engineering rationally heterostructures toward high-performance energy storage devices.

## Experimental Section

### Material Preparation

Synthesis of the Prussian blue (PB) nanocubes. PB nanocubes were synthesized by a hydrothermal method reported previously with modifications [37]. In a typical process, 12 g polyvinylpyrrolidone (PVP, MW ≈ 40,000) and 0.6 g potassium hexacyanoferrate (II) (K_4_Fe(CN_6_)·3H_2_O) were added into 0.1 M hydrochloric acid (HCl, 37%) under magnetic stirring, to form a clear solution. Subsequently, the solution was transferred in a programmable oven and then heated to 80 °C for 24 h. Finally, PB nanocubes were obtained by centrifuging and washing with water and ethanol and then dried in a vacuum oven overnight.

Synthesis of nitrogen-doped porous nanocubic structure (FeCN). FeCN nanocubes were obtained by calcining the as-synthesized PB nanocubes at 500 °C for 4 h under argon flow with a heating rate of 1 °C min^−1^.

Synthesis of FeCN/MoS_2_ nanocomposite. Twenty-five milligrams of FeCN nanocubes was dispersed in a mixed solvent containing 15 mL dimethylformamide and 15 mL ethanol under ultrasonication for 30 min. Then, 50 mg of ammonium tetrathiomolybdate ((NH_4_)_2_MoS_4_) was added to the above suspension under magnetic stirring. After stirring for 12 h, the mixture was transferred into a Teflon-lined stainless-steel autoclave and heated at 210 °C for 10 h. Then, the autoclave was cooled to room temperature naturally. The obtained FeCN/MoS_2_ nanocomposite was centrifuged and washed with water and ethanol for several times, and dried in a vacuum oven at 80 °C overnight.

Synthesis of Fe_1−*x*_S/MoS_2_ nanocomposite. The as-prepared FeCN/MoS_2_ composite and sodium hydrosulfide (NaHS·H_2_O) as sulfurization precursor were placed in an alumina boat that was inserted into a tubular furnace. Subsequently, the tubular furnace was heated to 400 °C for 4 h and then heated to 600 °C for 2 h under argon flow with a heating rate of 2 °C min^−1^. Finally, Fe_1−*x*_S/MoS_2_ nanocomposite was obtained.

For comparison, bare Fe_1−*x*_S nanocubes were synthesized through the same method without (NH_4_)_2_MoS_4_ in similar conditions.

### Material Characterization

Crystal structure of all samples was performed by X-ray diffraction techniques (XRD, Bruker D8 Advance) with Cu K*α* radiation operated at 40 kV and 25 mA. The morphology and microstructure were observed using field emission scanning electron microscope (FESEM, JEOL JSM-7600F), transmission electron microscopy and high-resolution transmission electron microscopy (TEM and HRTEM, JEOL JEM-2010). The surface electronic states were analyzed by X-ray photoelectron spectroscopy (XPS, PHI Quantera II) with Al K*α* source operated at 1486.6 eV. Nitrogen adsorption–desorption isotherms were carried out on a Quantachrome Autosorb-IQ analyzer. For in situ XRD measurement, each scan was collected between 10° and 50° using an electrochemical cell module equipped with metal beryllium (Be) and carbon paper as the window and current collector, respectively.

### Electrochemical Measurements

To fabricate working electrodes, active materials (Fe_1−*x*_S/MoS_2_ or Fe_1−*x*_S), acetylene black and carboxymethyl cellulose (CMC) at a weight ratio of 7:2:1 were dispersed in deionized water to form a homogeneous slurry. The resultant slurry was coated on the copper foil and then dried at 120° for 12 h in a vacuum oven. In a typical assembly process of CR2032 coin cells, sodium foil was used as the reference and counter electrodes, and glass fibers (GF, Whatman) were used as separators. 1 M NaPF_6_ was dissolved into a mixed solution of ethylene carbonate (EC) and diethyl carbonate (DEC) (EC/DMC, 1:1/v: v) with 2.5 wt% fluoroethylene carbonate (FEC) as electrolyte. The galvanostatic charge/discharge tests were conducted on a battery testing system (Neware) at different densities in a voltage range of 0.01–3.0 V. Cyclic voltammetry (CV) was carried out using an electrochemical workstation (VMP3, Bio-Logic) at different scan rates. Electrochemical impedance spectroscopy (EIS) was performed on the same workstation in a frequency range from 100 kHz to 10 mHz.

### DFT Calculations

All the calculations are based on density functional theory (DFT) using the plane-wave pseudopotentials [38, 39] with exchange–correlation of Perdew–Burke–Ernzerhof (PBE) [40, 41] formation as implemented in the Vienna Ab initio Simulation Package (VASP) [42]. A cutoff energy of 450 eV is employed for the plane-wave expansion of the wave functions. The Brillouin zone is sampled with 3 × 3 × 1 Monkhorst–Pack *k*-point mesh [43] for the structural optimization. The convergence criteria for the total energy and ionic forces were set to 10^−4^ eV and 0.1 eV Å^−1^, respectively. The construction with a 20 Å vacuum zone in the z direction to minimize the interactions between adjacent images. The climbing-image nudged elastic band (NEB) method [44] is used to seek the minimum energy pathways and determine the diffusion energy barriers.

## Results and Discussion

**Fig. 1:**
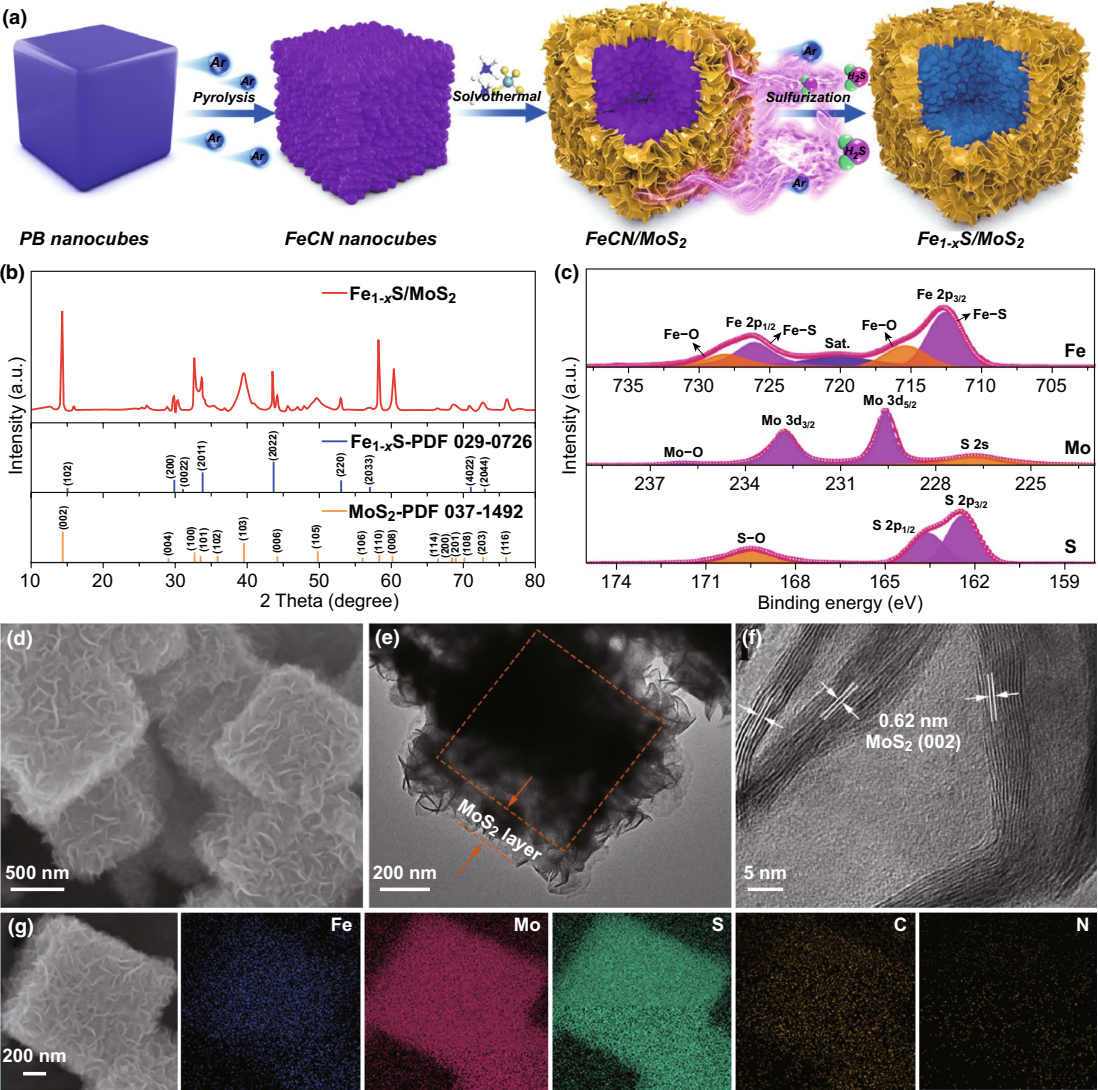
**a** Schematic illustration of the synthesis process for Fe_1−*x*_S/MoS_2_ nanocomposite. **b** XRD pattern and **c** high-resolution XPS spectra (Fe 2*p*, Mo 3*d*, and S 2*p*) of Fe_1−*x*_S/MoS_2_ composite. **d** SEM, **e** TEM and **f** HRTEM images of Fe_1−*x*_S/MoS_2_ composite. **g** Element mappings of the Fe_1−*x*_S/MoS_2_ composite

The synthetic procedures for the Fe_1−*x*_S/MoS_2_ nanocomposite are illustrated in Fig. 1a. PB nanocubes were first synthesized using a simple hydrothermal method. The as-prepared PB shows the typical diffraction peaks ascribed to Fe_4_[Fe(CN_6_)]_3_ with a face-centered-cubic structure (JCPDS NO. 73-0687) and a well-defined nanocubic morphology with an average size of about 700 nm (Fig. S1a, b). Next, PB nanocubes were pyrolyzed to nitrogen-doped porous nanocubic structure (FeCN) nanocubes in an inert atmosphere. As shown in Fig. S1c, the cubic framework is well maintained. MoS_2_ nanosheets were then uniformly grown on surface of FeCN nanocubes, resulting in an enlarged nanocube size of about 1 μm (Fig. S1d). Finally, Fe_1−*x*_S/MoS_2_ composite was obtained without obvious structural change after a sulfurization process (Fig. 1d). Figure 1b shows XRD pattern of the as-synthesized Fe_1−*x*_S/MoS_2_ composite. The diffraction peaks at 30.3°, 33.7°, 43.5° and 53.0° are assigned to the (200), (2011), (2022) and (220) planes of hexagonal pyrrhotite Fe_1−*x*_S (JCPDS NO. 29-0726), respectively. Besides, a series of strong peaks at 14.3°, 29.8°, 32.7°, 39.5°, 44.2°, 49.7°, 58.2° and 60.3° can be readily indexed to the (002), (004), (100), (103), (006), (105), (110) and (008) planes of MoS_2_ (JCPDS No. 37-1492). XPS analysis was performed to investigate the chemical compositions and surface electronic states of Fe_1−*x*_S/MoS_2_ composite. The survey spectra confirm the presence of Fe, Mo, S, C, and N elements (Fig. S2). In the Fe 2*p* XPS spectrum, the peaks located at 712.7 and 726.2 eV are corresponded to the Fe-S bonds along with a corresponding satellite at 720.3 eV, whereas the observed peaks at 715.5 and 728.2 eV indicate the presence of Fe–O bonds (Fig. 1c) [45]. In the S 2*p* XPS spectrum, peaks at 162.5 and 163.7 eV are correlated to the S 2*p*_1/2_ and S 2*p*_3/2_, respectively, while the peak centered at 169.4 eV is attributed to S–O bond from superficial oxidized sulfur species [46]. The Mo 3*d* XPS spectrum shows two obvious peaks at 229.6 and 232.8 eV corresponding to Mo 3*d*_3/2_ and Mo 3*d*_5/2_, respectively, which are the characteristic of Mo^4+^, whereas the peak at 236.2 eV is attributed to Mo–O bond [47]. TEM image confirms that MoS_2_ nanosheets are well anchored on the Fe_1−*x*_S nanocubes, and there is an obvious interface between the Fe_1−*x*_S and MoS_2_ (Fig. 1e). The average thickness of MoS_2_ is revealed to be around 150 nm, well consistent with SEM observation. High-resolution TEM image shows well-resolved lattice fringes with *d*-spacing of 0.62 and 0.23 nm, corresponding to the (002) and (103) planes of MoS_2_, respectively (Fig. 1f). No obvious lattice fringes of Fe_1−*x*_S can be observed due to the coverage of thick MoS_2_ nanosheets. EDS elemental mapping images manifest the existence of Fe, Mo, S, C, and N elements, further confirming the successful synthesis of Fe_1−*x*_S/MoS_2_ heterostructure (Fig. 1g). The existence of C and N elements could be attributed to the formation of N-doped carbon, which is expected to enhance the electronic conductivity of electrode materials [48, 49]. For comparison, bare Fe_1−*x*_S nanocubes were also synthesized by the same route without MoS_2_. As shown in Fig. S3a, all diffraction peaks of Fe_1−*x*_S could be ascribed to the hexagonal pyrrhotite Fe_1−*x*_S (JCPDS NO. 29-0726) without any other impurity phases. SEM and TEM images show that the nanocubic-shaped morphology is well retained with a much rougher surface and porous structure (Fig. S3b-e). HRTEM image clearly displays the lattice spacing of 0.29 nm, corresponding to (200) plane of Fe_1−*x*_S (Fig. S3f). In addition, the nitrogen adsorption–desorption isotherms in Fig. S4 indicate that Fe_1−*x*_S/MoS_2_ composite possesses higher BET surface area of 45.6 m^2^ g^−1^ than that of pure Fe_1−*x*_S (12.5 m^2^ g^−1^) (Fig. S4). The composite structures with high porosity can provide abundant contact area and diffusion channels for electrolyte.

**Fig. 2:**
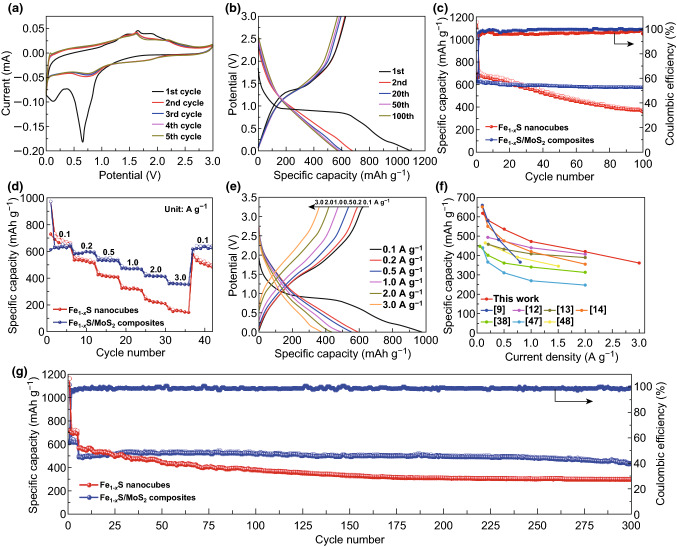
**a** CV curves of Fe_1−*x*_S/MoS_2_ composite electrode for the first five cycles. **b** Galvanostatic charge–discharge profiles of Fe_1−*x*_S/MoS_2_ composite electrode at 100 mA g^−1^. **c** Cycling performance at 100 mA g^−1^. **d** Rate capability at various current densities. **e** Galvanostatic charge–discharge profiles of Fe_1−*x*_S/MoS_2_ composite electrode at various current densities. **f** The comparison of the rate capability with other iron sulfide-based anode materials previously reported [9, 12–14, 38, 55, 56]. **g** Long-term cycling performance at 1.0 A g^−1^

Figure 2a shows the CV curves of Fe_1−*x*_S/MoS_2_ heterostructure for the first five cycles. During the first discharge process, three obvious cathodic peaks at around 1.10, 0.60, and 0.15 V can be assigned to the multistep sodiation processes involving the intercalation of Na^+^ into Fe_1−*x*_S/MoS_2_ and the formation of Mo^0^/Fe^0^ [13, 35, 50]. Meanwhile, the strong peak at about 0.60 V is also associated with the formation of SEI films, and the peak intensity decreases obviously in the subsequent cycles [38, 51]. For the anodic scan, two obvious peaks at about 1.60 and 1.90 V correspond to the stepwise desodiation process [52]. From the second cycle onward, the reversible reactions of Na_2-*y*_Fe_1−*x*_S_2_/Na_2_Fe_1−*x*_S_2_ and MoS_2_/Mo^0^ enable the reversible Na storage in composite electrode [12, 53]. These peaks related to the phase transformation during sodiation/desodiation process will be further discussed on the basis of in situ XRD analysis. The CV curves of Fe_1−*x*_S/MoS_2_ composite electrode basically overlap from the second cycle onward, indicating excellent electrochemical reversibility and structural stability of the heterostructure. In contrast, bare Fe_1−*x*_S nanocubes show inferior electrochemical reversibility (Fig. S5).

Figure 2b presents the galvanostatic charge–discharge curves of Fe_1−*x*_S/MoS_2_ composite electrode under different cycles at 100 mA g^−1^. The Fe_1−*x*_S/MoS_2_ composite exhibits an initial discharge and charge capacities of 1072.1 and 645.0 mAh g^−1^, respectively, showing an initial Coulombic efficiency of 60%. The irreversible capacity loss arises from the formation of SEI layer on the electrode surface [54]. However, the bare Fe_1−*x*_S electrode delivers a lower initial Coulombic efficiency of 58% at the same current density (Fig. S6). After 100 cycles, the Fe_1−*x*_S/MoS_2_ heterostructure demonstrates a high reversible capacity of 584.7 mAh g^−1^ with capacity retention of approximately 91%, and high Coulombic efficiency from the second cycle onward (Fig. 2c). In contrast, the bare Fe_1−*x*_S shows rapid capacity decay and lower Coulombic efficiency during the whole process.

The superiority of the heterostructure is also highlighted by its outstanding rate capability. The Fe_1−*x*_S/MoS_2_ composite electrode delivers the average reversible specific capacities of 637.2, 594.7, 552.8, 490.4, 432.3, and 372.1 mAh g^−1^ at the current densities of 0.1, 0.2, 0.5, 1.0, 2.0, and 3.0 A g^−1^, respectively. When the current density returns to 0.1 A g^−1^, the specific capacity can revert back to a high value of 636.7 mAh g^−1^ with approximately 100% capacity retention (Fig. 2d, e). Such excellent rate capability is superior to most of other iron sulfide-based anode materials reported previously, as shown in Fig. 2f. However, with the increasing current density, the bare Fe_1−*x*_S electrode shows a much lower capacity (Fig. S7). Furthermore, the long-term cycling performance of the Fe_1−*x*_S/MoS_2_ electrode was evaluated at 1.0 A g^−1^ (Fig. 2g) with an initial five cycles activation at 0.1 A g^−1^. After 300 cycles, a high reversible capacity of 396.8 mAh g^−1^ is achieved, implying the superior high-rate cycling stability. However, the bare Fe_1−*x*_S electrode shows rather poor cycle performance under the identical testing conditions. *Ex situ* SEM was then conducted after cycling. As shown in Fig. S8, Fe_1−*x*_S/MoS_2_ composite almost maintains the structural integrity without obvious damage, confirming the good structural stability for sodium storage. Such excellent cycling and rate performance might be related to the following unique hetero-nanostructure design advantages. First, the porous MoS_2_ nanosheets enlarge the electrode/electrolyte contact area and reduce the charge-transfer resistance along the electrode/electrolyte interface. Second, the heterostructure structure design prevents the nanocubes agglomeration and accommodates the severe structural deformation significantly [35]. More importantly, the heterointerface between the Fe_1−*x*_S and MoS_2_ could serve as an “ion reservoir” to significantly boost Na^+^ capture/storage and then accelerate the Na^+^ diffusion from the shell to the internal part [31].

**Fig. 3:**
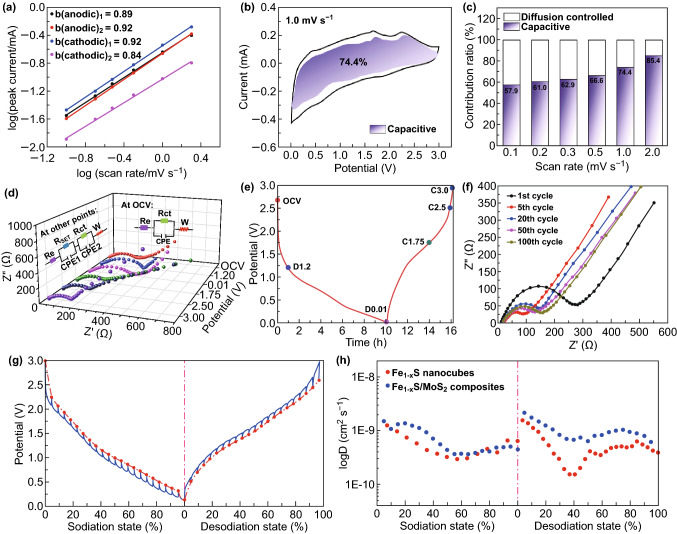
Kinetic analysis of sodium storage behavior for Fe_1−*x*_S/MoS_2_ composite. **a** Linear relationship between log (*i*) and log (*v*). **b** Capacitive and diffusion-controlled contribution at 1.0 mV s^−1^. **c** Normalized contribution ratio of capacitive capacities at different scan rates. **d** In situ EIS spectra evolution at different charge/discharge potentials. **e** First charge/discharge profile at 100 mA g^−1^ with labeled points for EIS. **f** EIS spectra after different cycles. **g** GITT curve. **h** The corresponding Na^+^ diffusion coefficient at different discharge/charge state of Fe_1−*x*_S/MoS_2_ composite and pure Fe_1−*x*_S electrodes

To further interpret the outstanding sodium storage performance of Fe_1−*x*_S/MoS_2_ heterostructure, kinetics analysis based on CV measurements at different scan rates was carried out. As shown, the CV curves from 0.1 to 1.0 mV s^−1^ exhibit similar shape with broad reduction/oxidation peaks related to the sodiation/desodiation processes (Fig. S9). The relationship between the peak current (*i*) and scan rate (*v*) obeys the power law as shown in Eq. (1) [57]:

1 $${\text{i}} = {\text{av}}^{b}$$

where both *a* and *b* are constants. The *b* value reveals the charge storage mechanism (*b *= 0.5 represents a diffusion-controlled process; *b *= 1.0 refers to a surface capacitance-controlled process). As shown in Fig. 3a, the *b* values obtained from the log (*v*) versus log (*i*) plots for peak 1, 2, 3, and 4 are 0.84, 0.92, 0.89, and 0.92, respectively, suggesting the charge storage in Fe_1−*x*_S/MoS_2_ composite is dominated by surface capacitive behavior.

To be more intuitive, the quantitative pseudocapacitance contribution at different scan rates could be calculated according to Eq. (2) [58]:

2 $${\text{i}}\left( V \right) = k_{1} v + k_{2} v^{1/2}$$

where *k*_1_ and *k*_2_ are constants for a fixed voltage. The *k*_1_*v* indicates the current response from surface capacitive contribution, while *k*_2_*v*^1/2^ represents the diffusion-controlled current. The surface capacitive contribution of the Fe_1−*x*_S/MoS_2_ composite electrode at 1.0 mV s^−1^ is about 74.4% as displayed by the shaded area (Fig. 3b). With the increase in scan rate, the pseudocapacitance contribution gradually increases from 57.9% to 85.4% (Fig. 3c). However, the bare Fe_1−*x*_S electrode shows a much lower capacitive contribution at different scan rates (Fig. S8). The higher pseudocapacitance contribution for Fe_1−*x*_S/MoS_2_ composite determines a more favorable electrochemical kinetics behavior at high current density, which is consistent with its excellent rate capability as shown in Fig. 2.

In situ electrochemical impedance spectroscopy (EIS) during the first sodiation/desodiation process was conducted to investigate the kinetics differences induced by structural and phase evolution at different discharge/charge states. Figure 3d shows the EIS behavior of Fe_1−*x*_S/MoS_2_ composite in the first cycle with labeled points presented in Fig. 3e. All Nyquist plots show similar features with a depressed semicircle in high–medium-frequency region and an oblique line in low-frequency region. Based on the fitted equivalent circuits, at open-circuit voltage (OCV) state, the semicircle can be ascribed to the charge-transfer resistance (*R*_ct_) and the oblique line represents the Warburg impedance (*W*) related to the Na^+^ diffusion [59]. However, at other states, the semicircle corresponds to two overlapping parts of the SEI film resistance (*R*_SEI_) and *R*_ct_ [60]. During the sodiation process, due to the formation of insulative Na_2_S matrix and SEI films accompanied by the gradual volume change, the resistance increases obviously from OCV to 0.01 V [61]. During the desodiation process, the nanoclustered Na_*y*_Fe_1−*x*_S_2_ phase and metallic state 1T-MoS_2_ gradually form, and meanwhile, non-conductive Na_2_S gradually disappears. As a consequence, the resistance decreases gradually from 0.01 to 3.0 V. In addition, EIS measurements were also performed after different cycles at current density of 100 mA g^−1^ to show the charge-transfer stability. With the increase in cycles, the resistance of Fe_1−*x*_S/MoS_2_ composite electrode decreases initially owing to the activation process and then slightly increases after several cycles (Fig. 3f). In contrast, Nyquist spectra of the bare Fe_1−*x*_S electrode show higher resistances at different cycles. Overall, the in situ EIS measurements have confirmed the enhanced charge-transfer kinetics and electronic conductivity of the Fe_1−*x*_S/MoS_2_ composite (Fig. S10) [62, 63].

Galvanostatic intermittent titration technique (GITT) was further performed to investigate the influence of multistep sodiation and desodiation reactions of Fe_1−*x*_S/MoS_2_ heterostructure on Na-ion chemical diffusion coefficient (*D*_*Na*_) (Figs. 3g and S12). From Fig. 3h, the Na^+^ diffusion coefficient fluctuates with the progress of sodiation/desodiation, and the minimum values appear at each cathodic/anodic reaction plateau, where Na ions diffuse deeply into/from the internal crystal structure. Obviously, the Fe_1−*x*_S/MoS_2_ composite electrode shows a higher diffusion coefficient and minor change than those of bare Fe_1−*x*_S, which can be attributed to the unique heterointerface to act as an “ion reservoir” and fast diffusion channel for Na ions. In addition, phase boundaries can suppress the growth of crystal domains, thus forming numerous defects and active sites to facilitate Na^+^ diffusion [64]. Furthermore, benefiting from out-of-sync electrochemical reactions of these two sulfides at different voltages, the structural stress could be effectively mitigated, which is also favorable for diffusion of sodium ions.

**Fig. 4:**
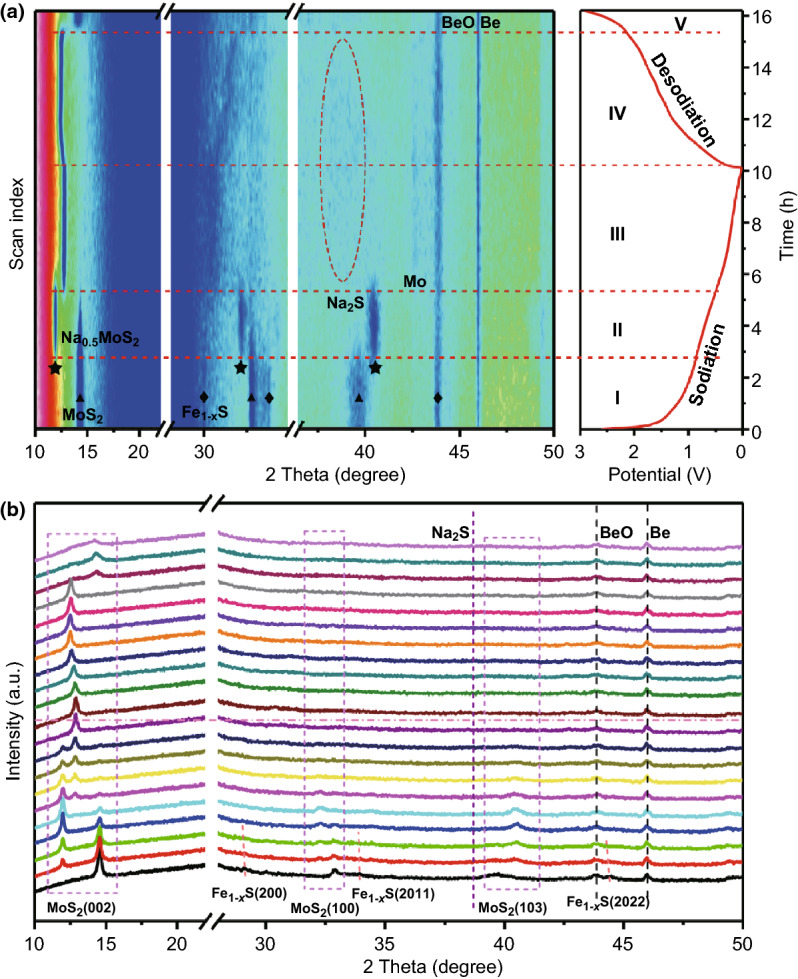
**a** Contour plots of in situ XRD results and **b** selected diffraction patterns of Fe_1−*x*_S/MoS_2_ composite electrode during the first sodiation/desodiation process

To further reveal the voltage-dependent phase transformation behavior of Fe_1−*x*_S/MoS_2_ heterostructure during the first sodiation/desodiation process, in situ XRD was performed between 0.01 and 3.0 V at the current density of 80 mA g^−1^ (Fig. 4). Noticed that the strong peaks located at about 26.7°, 44.0°, and 46.0° are derived from carbon paper, BeO, and Be, respectively. As shown in Fig. 4, the peaks at 29.9°, 33.7°, and 43.5° are related to the (200), (2011), and (2022) planes of Fe_1−*x*_S, and the peaks at about 14.3°, 32.7°, and 39.5° correspond to the (002), (100), and (103) diffractions of MoS_2_. During the sodiation process, the peaks of Fe_1−*x*_S gradually shift to a lower 2*θ*, suggesting the formation of intermediate Na_2_Fe_1−*x*_S_2_ through the Na^+^ insertion into Fe_1−*x*_S. When continuously discharging to 0.01 V, the peaks related to Na_2_Fe_1−*x*_S_2_ gradually disappear, along with the gradual increase in the diffraction peak corresponding to Na_2_S. Fe peaks could not be clearly observed in the process, which is mostly due to either the ultra-small crystal size or amorphous nature of resultant Fe^0^ [65]. On the other hand, the prominent peak at 14.3° shifts obviously during the whole sodiation/desodiation process, indicating the structural changes of MoS_2_. In stages I and II, there are two stages involving two-phase transitions: One is from 2H-MoS_2_ (2*θ*_(002)_ = 14.3°) to 2H-Na_0.5_MoS_2_ (2*θ*_(002)_ = 11.8°) and the other is from 2H-Na_0.5_MoS_2_ to 1T-NaMoS_2_ (2*θ*_(002)_ = 12.6°) [66]. Subsequently, more Na ions insertion into Na_*z*_MoS_2_ did not induce any detectable phase changes. In stage III, the intensity of diffraction peak related to Na_2_S gradually increases accompanied by the formation of Mo. After recharging back to 1.6 V, the (002) main peak gradually shifts toward a lower 2*θ* angle to form 1T-NaMoS_2_. In subsequent deintercalation process, the diffraction peak shifts back to a higher 2*θ* angle with a two-phase transition from 1T-Na_0.5_MoS_2_ to 1T-MoS_2_ (2*θ*_(002)_ = 14.1°) rather than the initial 2H-MoS_2_ (2*θ*_(002)_ = 14.3°) [47, 67]. Meanwhile, the diffraction peaks corresponding to Na_2_S and Mo gradually disappear. Interestingly, the diffraction peak related to Na_*z*_MoS_2_ always exists in the whole conversion reaction process, implying the incomplete conversion from Na_*z*_MoS_2_ to Mo, which is favorable for structural stability. At the desodiation process, due to the strong peak of carbon paper, the peaks at around 30° could not be clearly assigned to the Na_2_Fe_1−*x*_S_2_ and Na_2-*y*_Fe_1−*x*_S_2_, which are similar to the previous report for iron sulfide materials [12]. Therefore, from the above in situ XRD and CV analysis, the multistep reaction mechanisms for Fe_1−*x*_S/MoS_2_ heterostructure at different sodiation/desodiation states could be expressed as follows:

Sodiation process:

$$\begin{aligned} {\text{Stage I }}\left( {\text{intercalation}} \right) & :2{\text{Fe}}_{1 - x} {\text{S }} + 2{\text{Na}}^{ + } + 2{\text{e}}^{ - } \to {\text{ Na}}_{2} {\text{Fe}}_{1 - x} {\text{S}}_{2} + \, \left( {1 - x} \right){\text{Fe}} \\ &\quad 2{\text{H}} - {\text{MoS}}_{2} + 0.5{\text{Na}}^{ + } + \, 0.5{\text{e}}^{ - } \to \, 2{\text{H}} - {\text{Na}}_{0.5} {\text{MoS}}_{2} \\ \end{aligned}$$

$$\begin{aligned} {\text{Stage II }}\left( {\text{intercalation}} \right): & 2{\text{H}} - {\text{Na}}_{0.5} {\text{MoS}}_{2} + 0.5{\text{Na}}^{ + } + 0.5{\text{e}}^{ - } \to \, 1{\text{T}} - {\text{NaMoS}}_{2} \\ &\quad 1{\text{T}} - {\text{NaMoS}}_{2} + \left( {z - 1} \right){\text{Na}}^{ + } + \left( {z - 1} \right){\text{e}}^{ - } \to \, 1{\text{T}} - {\text{Na}}_{z} {\text{MoS}}_{2} \\ \end{aligned}$$

$$\begin{aligned} {\text{Stage III }}\left( {\text{conversion}} \right): & 1{\text{T}} - {\text{Na}}_{z} {\text{MoS}}_{2} + \, \left( {4 - z} \right){\text{Na }} + \, \left( {4 - z} \right){\text{e}}^{ - } \to {\text{Mo}} + \, 2{\text{Na}}_{2} {\text{S}} \\ &\quad{\text{Na}}_{2} {\text{Fe}}_{1 - x} {\text{S}}_{2} + \, 2{\text{Na }} + 2{\text{e}}^{ - } \to \, \left( {1 - x} \right){\text{Fe }} + \, 2{\text{Na}}_{2} {\text{S}} \\ \end{aligned}$$

Desodiation process:

$$\begin{aligned} {\text{Stage IV }}({\text{deconversion}}): & \, \left( {1 - x} \right){\text{Fe }} + \, 2{\text{Na}}_{2} {\text{S }} \to {\text{Na}}_{2} {\text{Fe}}_{1 - x} {\text{S}}_{2} + \, 2{\text{Na}} + 2{\text{e}}^{ - } \\ &\quad{\text{Mo }} + \, 2{\text{Na}}_{2} {\text{S }} \to \, 1{\text{T}} - {\text{Na}}_{0.5} {\text{MoS}}_{2} + \, 3.5{\text{Na}} + 3.5{\text{e}}^{ - } \\ \end{aligned}$$

$$\begin{aligned} {\text{Stage V }}\left( {\text{deintercalation}} \right): & {\text{Na}}_{2} {\text{Fe}}_{1 - x} {\text{S}}_{2} \to {\text{ Na}}_{2 - y} {\text{Fe}}_{1 - x} {\text{S}}_{2} + y{\text{Na}} + y{\text{e}}^{ - } \\ &\quad1{\text{T}} - {\text{Na}}_{0.5} {\text{MoS}}_{2} \to \, 1{\text{T}} - {\text{MoS}}_{2} + \, 0.5{\text{Na}} + \, 0.5{\text{e}}^{ - } \\ \end{aligned}$$

Furthermore, the structural change of Fe_1−*x*_S/MoS_2_ composite electrode at a high current density was also monitored by in situ XRD. As shown in Fig. S13, from the second cycle forward, the (002) diffraction peak exhibits small periodic change, confirming the stable framework and kinetic processes of this composite structure during cycling.

**Fig. 5:**
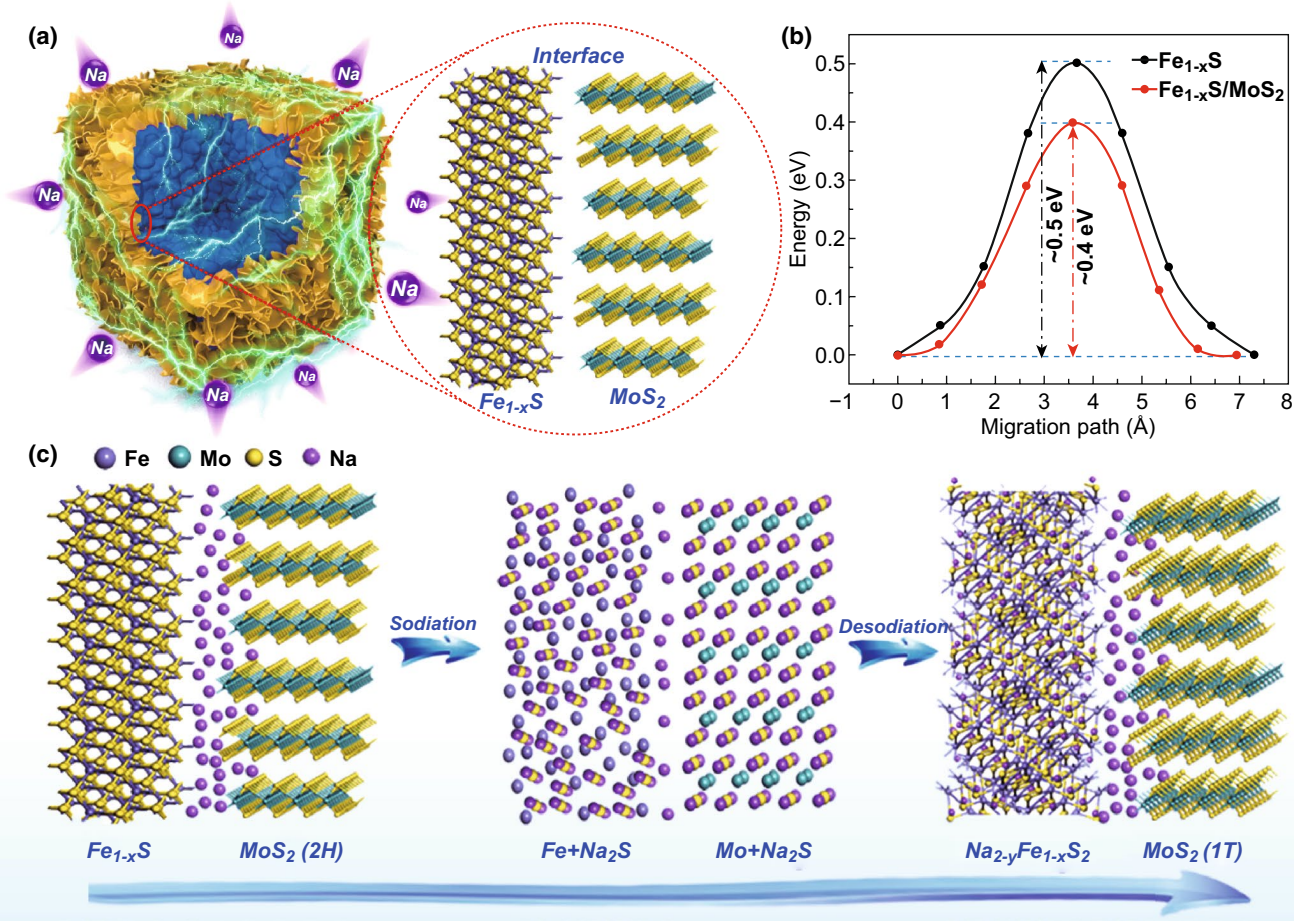
**a** Schematic illustration of Fe_1−*x*_S/MoS_2_ interface. **b** The diffusion barrier profiles of Na on Fe_1−*x*_S surface and Fe_1−*x*_S/MoS_2_ interface. **c** Proposed schematic atomistic models of the illustrated reaction mechanism for Fe_1−*x*_S/MoS_2_ heterostructure during the sodiation and desodiation process

DFT calculations were carried out to investigate the heterointerfacial behavior to present atomic-level verification for the superior sodium storage capability of the heterostructure. The Na^+^ diffusion barriers in bare Fe_1−*x*_S and Fe_1−*x*_S/MoS_2_ heterostructure were calculated (Figs. 5b and S14). As shown, the barrier is about 0.4 eV in MoS_2_ side close to the interface for heterostructure, obviously lower than that in bare Fe_1−*x*_S (~ 0.5 eV). This result indicates that Na^+^ migration is more favorable in Fe_1−*x*_S/MoS_2_ heterointerface, which is beneficial for the improvement of electrochemical reaction kinetics. On the basis of the above analysis, a mechanism for enhanced electrochemical performance was proposed as illustrated in Fig. 5c. The Fe_1−*x*_S/MoS_2_ heterostructure is composed of hexagonal Fe_1−*x*_S nanocubes and 2D layered MoS_2_ nanosheets. Once discharging, the unique “stacking card” nanostructures derived from the assembled MoS_2_ nanosheets can accelerate the electrolyte permeation and Na^+^ migration. Benefiting from the low diffusion barrier, plenty of Na ions could store at the interface, then forming the so-called ion reservoir. High concentration gradient also drives Na^+^ transport from the interface to the internal part, hence realizing an efficient conversion reaction kinetics [34]. After full sodiation, MoS_2_ is transformed into metallic Mo and Na_2_S. During the desodiation process, metallic Mo nanoclusters can serve as “pin conductor” until 1T-MoS_2_ phase is formed, dramatically improving the electrical conductivity of the whole electrode and then facilitating the conversion reaction [31]. In the subsequent cycles, such enhancement effects could be retained owing to the regeneration of heterointerface. The difference is that the original 2H-MoS_2_ phase was completely converted into 1T-MoS_2_ phase along with the change of electronic states between semiconductive and metallic, which can greatly enhance electrical conductivity, as well as boost Na^+^ diffusion and accelerate the charge-transfer kinetics [67]. Therefore, such outstanding sodium storage performance can be mainly attributed to the unique heterostructure design: (1) Low Na^+^ diffusion barrier originated from Fe_1−*x*_S/MoS_2_ heterointerface can significantly promote reaction kinetics; (2) the phase transformation from 2H- to 1T-MoS_2_ after the initial cycle can greatly enhance electrical conductivity; (3) nanoarchitectures enable shorten ion diffusion pathway and mitigate the volume change; (4) high specific surface area caused by hierarchical MoS_2_ nanosheet decoration is able to further buffer the structural stress and facilitate electrolyte permeation.

## Conclusion

In conclusion, we have demonstrated experimental and theoretical evidence that the electrochemical reaction kinetics could be significantly boosted by rationally designing heterostructure with abundant “ion reservoir” for sodium storage. The low diffusion barrier at the heterointerface greatly promotes sodium ion diffusion and charge-transfer kinetics. As a proof of concept, SIB anode based on Fe_**1−*x***_S/MoS_2_ heterostructure exhibits superior rate capability and long cycle life. In light of the analysis about the dynamic relationship between heterointerface and electrochemical performance, our present work provides a fundamental understanding on heterostructure engineering for high-performance energy storage devices.

## Electronic supplementary material

Below is the link to the electronic supplementary material.

Supplementary material 1 (PDF 1289 kb)
