# Supplementary material for: Boosting Sodium Storage of Fe1−xS/MoS2 Composite via Heterointerface Engineering
Source: Nanomicro Lett. 2019 Sep 23;11:80. doi: 10.1007/s40820-019-0311-z (PMC7770956; doi:10.1007/s40820-019-0311-z)
Supplement: Supplementary file 1 — Supplementary material 1 (PDF 1289 kb) [file 40820_2019_311_MOESM1_ESM.pdf]

Supporting Information for

Boosting Sodium Storage of $Fe_{1-x}S/MoS_2$ Composite via Heterointerface Engineering

Song Chen[1, 2], Shaozhuan Huang[2], Junping Hu[2], Shuang Fan[1, 2], Yang Shang[2], Mei Er Pam[2], Xiaoxia Li[2], Ye Wang[4], Tingting Xu[4], Yumeng Shi[1, 3, *], Hui Ying Yang[2, *]

[1]International Collaborative Laboratory of 2D Materials for Optoelectronics Science and Technology of Ministry of Education, College of Optoelectronic Engineering, Shenzhen University, Shenzhen 518060, People's Republic of China

[2]Pillar of Engineering Product Development, Singapore University of Technology and Design, 8 Somapah Road, 487372, Singapore

[3]Engineering Technology Research Center for 2D Material Information Function Devices and Systems of Guangdong Province, College of Optoelectronic Engineering, Shenzhen University, Shenzhen 518060, People's Republic of China

[4]Key Laboratory of Material Physics of Ministry of Education, School of Physics and Engineering, Zhengzhou University, Zhengzhou 450052, People's Republic of China

*Corresponding authors. E-mail: yanghuiying@sutd.edu.sg (Hui Ying Yang); yumeng_shi@163.com (Yumeng Shi)

Supplementary Figures

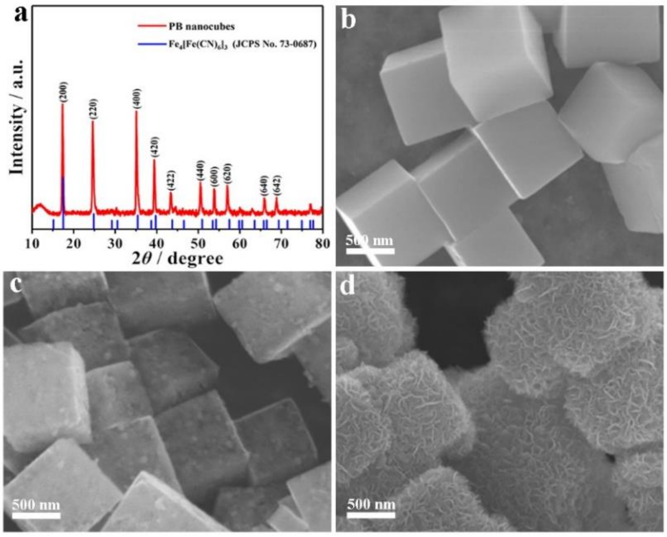

Fig. S1 a XRD pattern and **b** SEM image of PB nanocubes. SEM images of **c** FeCN nanocubes and **d** $FeCN/MoS_2$ composite

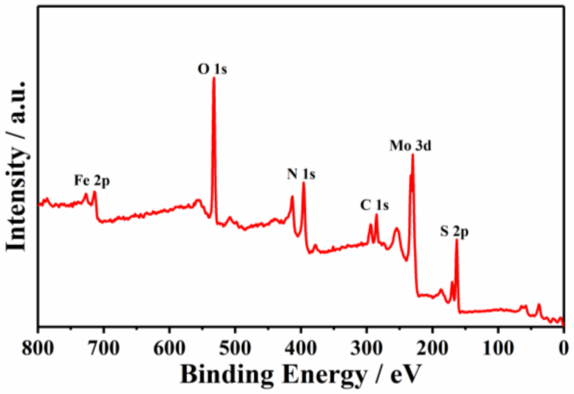

Fig. S2 Survey XPS spectra of the $Fe_{1-x}S/MoS_2$ composite

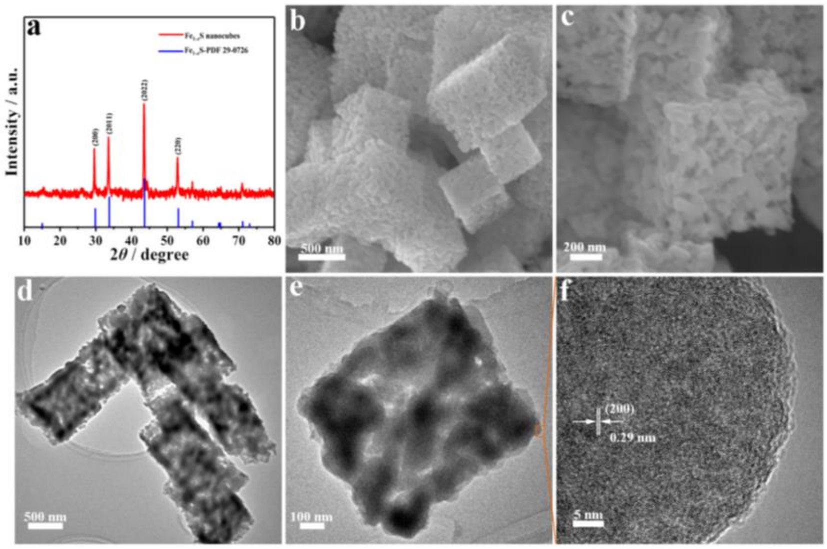

Fig. S3 **a** XRD pattern, **b, c** SEM images, **d, e** TEM images and **f** HRTEM image of $Fe_{1-x}S$ nanocubes

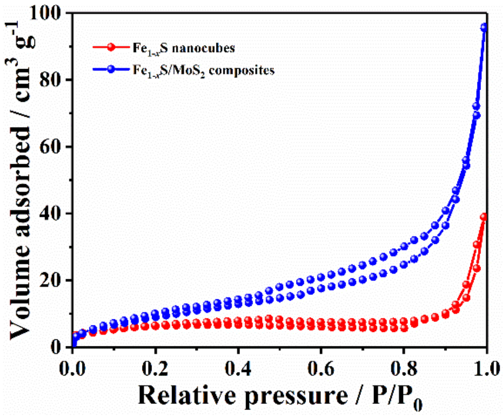

Fig. S4 Nitrogen adsorption-desorption isotherms of $Fe_{1-x}S/MoS_2$ composite/$Fe_{1-x}S$ nanocubes

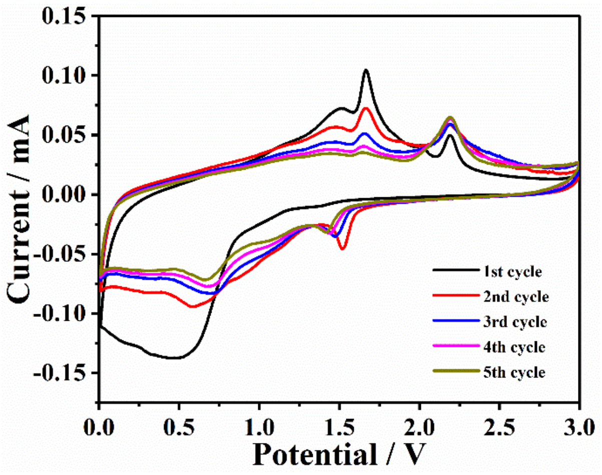

Fig. S5 CV curves of $Fe_{1-x}S$ nanocube electrode for the first five cycles

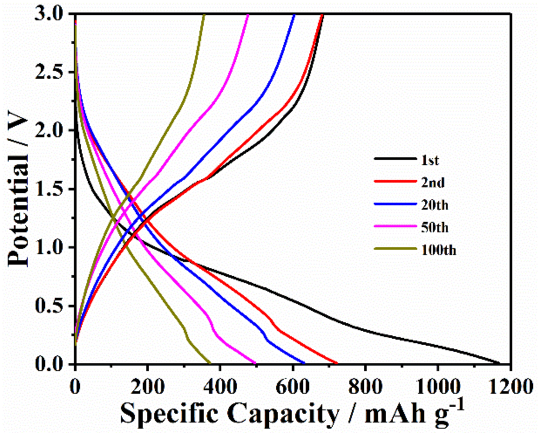

Fig. S6 Galvanostatic charge-discharge profiles of $Fe_{1-x}S$ nanocube electrode at 100 mA g^{-1}

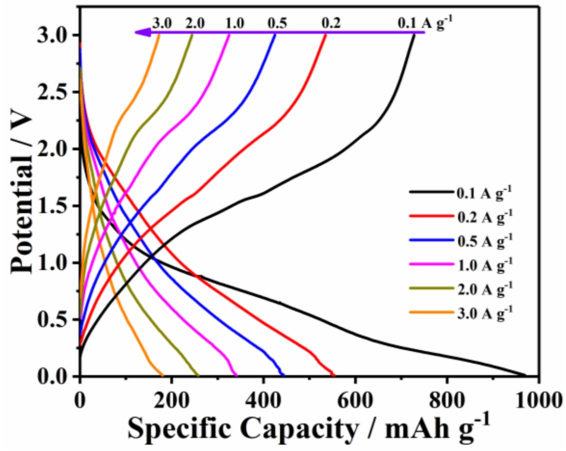

Fig. S7 Galvanostatic charge-discharge profiles of $Fe_{1-x}S$ nanocube electrode at various current densities

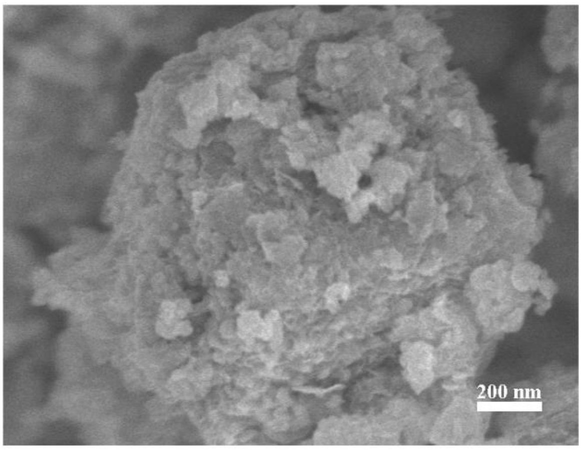

Fig. S8 SEM image of $Fe_{1-x}S/MoS_2$ composite after cycling

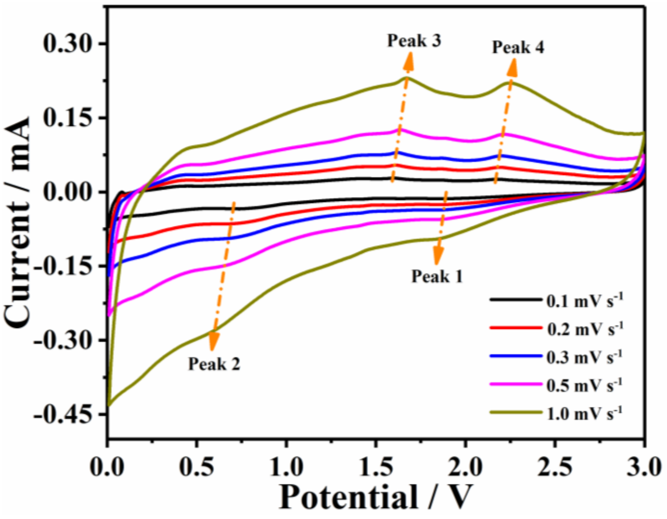

Fig. S9 CV curves of $Fe_{1-x}S/MoS_2$ composite at different scan rates

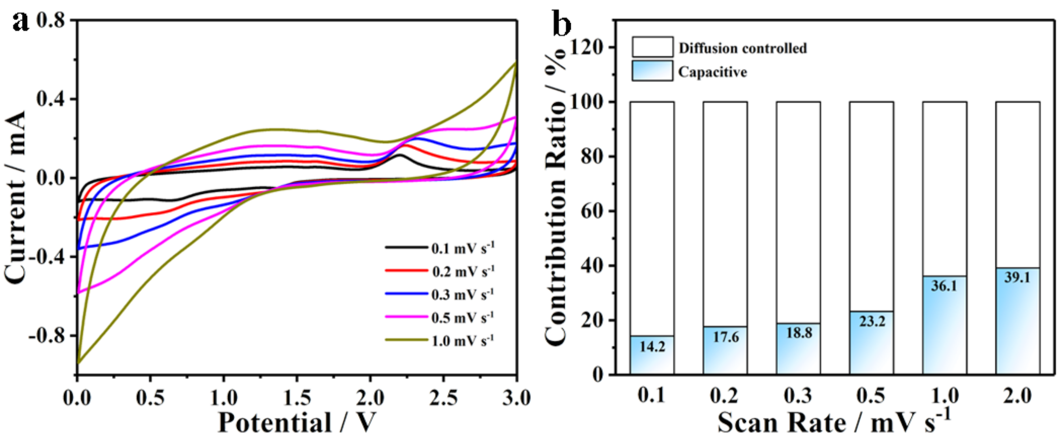

Fig. S10 a CV curves of $Fe_{1-x}S$ nanocubes at different scan rates. **b** Normalized contribution ratio of capacitive capacities at different scan rates

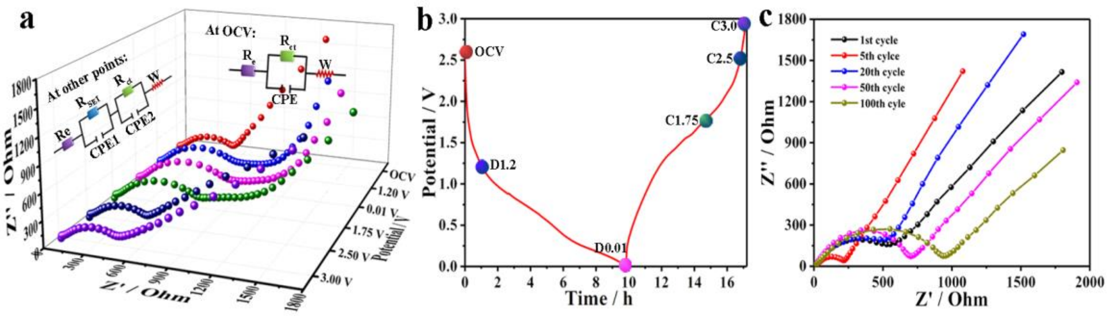

Fig. S11 a *In situ* EIS spectra evolution of $Fe_{1-x}S$ electrode at different charge/discharge potentials. **b** First charge/discharge profile of $Fe_{1-x}S$ electrode at 100 mA g^{-1} with labeled points for EIS. **c** EIS spectra of $Fe_{1-x}S$ electrode after different cycles

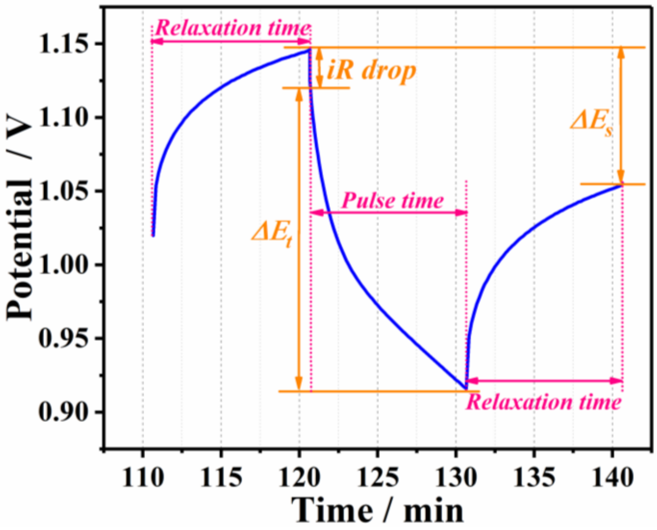

Fig. S12 E vs. t curve for a single GITT during discharge process

Na-ion chemical diffusion coefficient (D_{Na}) is calculated based on the following equation [S1]:

$$D = \frac{4L^2}{\pi\tau}\left(\frac{\Delta E_s}{\Delta E_t}\right)^2$$

where L is Na^+ diffusion length (approximately equal to the electrode thickness for compact electrode), τ is the relaxation time, ΔE_s is the steady state voltage change by the current pulse, ΔE_t is the voltage change during the current pulse after excluding *iR* drop.

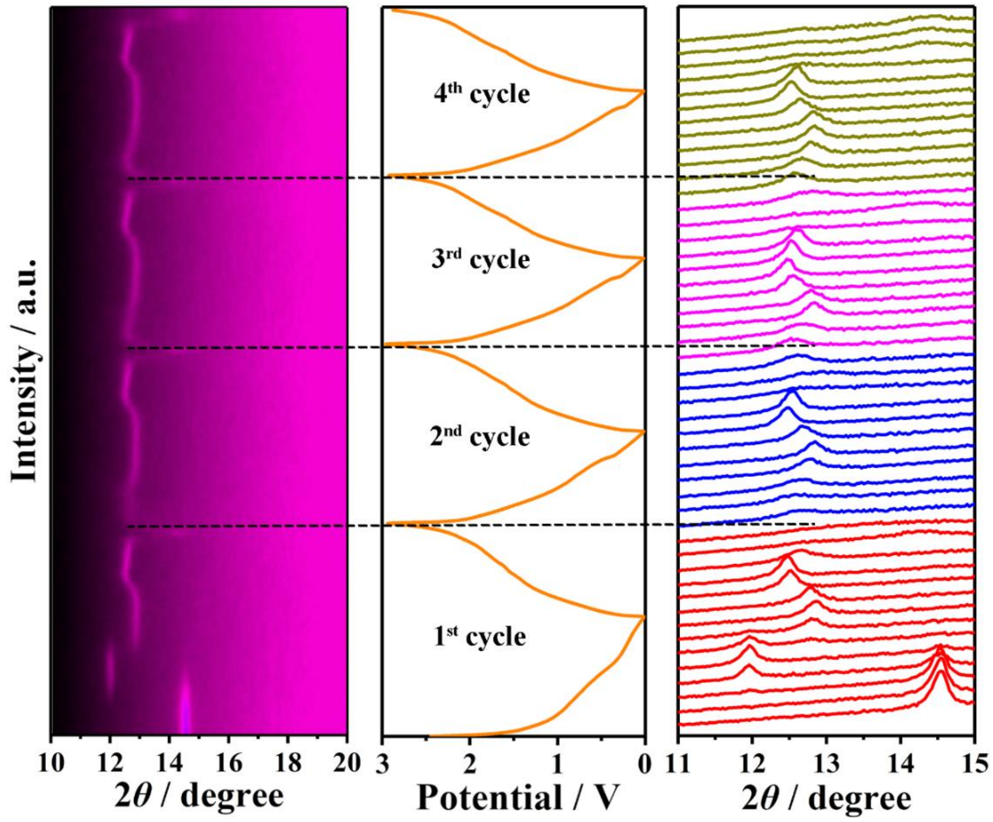

Fig. S13 Contour plots of *in situ* XRD results and the corresponding selected diffraction patterns of $Fe_{1-x}S/MoS_2$ composite electrode during the initial four cycles at 200 mA g^{-1}

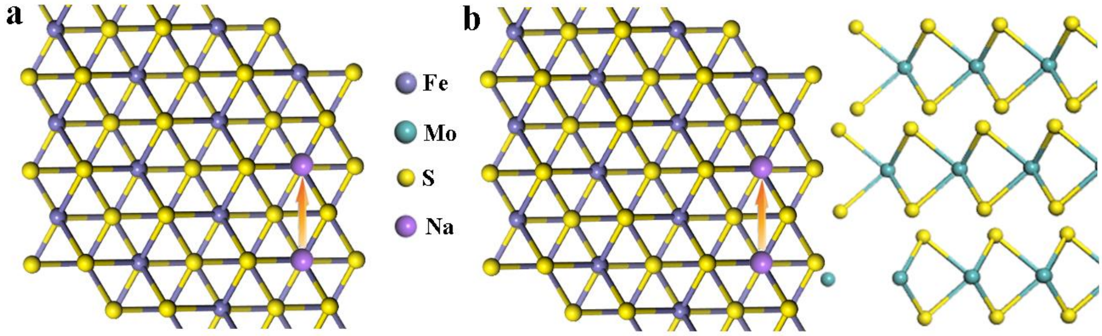

Fig. S14 The migration path on **a** $Fe_{1-x}S$ surface, and **b** $Fe_{1-x}S/MoS_2$ interface

Supplementary References

[S1]D.T. Ngo, H.T.T. Le, C. Kim, J.Y. Lee, J.G. Fisher, I.D. Kim, C.J. Park, Mass-scalable synthesis of 3D porous germanium–carbon composite particles as an ultra-high rate anode for lithium ion batteries. Energy Environ. Sci. **8**, 3577-3588 (2015). https://doi.org/10.1039/C5EE02183A
